# Impact of body mass index on outcomes in acute type A aortic dissection

**DOI:** 10.3389/fcvm.2026.1845403

**Published:** 2026-09-16

**Authors:** Adam H. Kiridly, Stevan S. Pupovac, Michele Dell'Aquila, Paley Arnone, Omar A. Jarral, Branden Tejada, Ian Mason, Robert Kalimi, S. Jacob Scheinerman, Alan R. Hartman, Derek R. Brinster

**Affiliations:** Department of Cardiothoracic Surgery, Northwell Health, New Hyde Park, NY, United States

**Keywords:** acute type A aortic dissection (ATAAD), mortality, obesity, risk, surgery

## Abstract

**Background:**

Elevated body mass index (BMI) is associated with adverse outcomes after cardiac surgery, but its impact in acute type A aortic dissection (ATAAD) surgery is unclear. We assessed the relationship between BMI and outcomes after ATAAD repair.

**Methods:**

An institutional retrospective cohort review was undertaken of all consecutive patients who underwent repair of ATAAD between July 2014 and December 2022 (*n* = 398). Patients were stratified by BMI into three groups: ‘non-obese’ [BMI < 30 kg/m^2^; median 26.0 (23.0, 27.7) kg/m^2^] (*n* = 248), ‘Obesity Class I’ [30 ≤ BMI < 35 kg/m^2^; median 31.9 (30.8, 33.4) kg/m^2^] (*n* = 81), and ‘Obesity Class II+’ [BMI ≥ 35 kg/m^2^; median 38.9 (36.4, 41.9) kg/m^2^] (*n* = 69). Multivariable logistic regression was utilized to assess the relationship between BMI and 30-day mortality, stroke, and duration of ventilation.

**Results:**

Thirty-day operative mortality for the entire cohort was 12.8% (*n* = 51). The Obesity Class I cohort was associated with decreased odds of 30-day mortality [OR = 0.18, 95% CI: (0.06–0.62), *p* = 0.006] compared to the non-obese cohort, whereas the Obesity Class II + was not. Diabetes [OR = 2.9, 95% CI: (1.32–6.41), *p* = 0.008], history of lower extremity ischemia [OR = 4.27, 95% CI: (2.11–8.62), *p* < 0.001], age [OR = 1.04, 95% CI: (1.01–1.07), *p* = 0.005], duration of cardiopulmonary bypass [OR = 1.005, 95% CI: (1.00–1.01), *p* = 0.027], and units of intraoperative red blood cells received [OR = 1.08, 95% CI: (1.01–1.16), *p* = 0.028] were associated with increased 30-day mortality. There was no significant association between BMI groups and post-operative stroke or duration of ventilation.

**Conclusion:**

The obesity paradox in cardiac surgery remains incompletely understood. In this cohort, BMI was not associated with increased 30-day mortality following ATAAD repair, with class I obesity demonstrating lower adjusted mortality. These findings support the continued practice of expeditious surgical intervention for ATAAD regardless of BMI.

## Introduction

Acute Stanford type A aortic dissection (ATAAD) is a life-threatening cardiovascular emergency requiring prompt surgical intervention (1–4). While diagnostic methods, surgical techniques, and peri-operative care have improved outcomes, significant morbidity and mortality continue to be associated with ATAAD.

Obesity is a disease that has long been associated with the development of cardiovascular pathology. Recent investigation into the inflammatory state, stress response, and changes in physiology associated with obesity has led many to question how obesity would affect outcomes after cardiac surgery (5, 6). While some have investigated the impact of obesity on outcomes after ATAAD repair, it has been incompletely studied, and therefore, we lack evidence-based guidelines for preoperative risk stratification and operative management of ATAAD in patients with obesity (1–4, 7, 8). Prior small studies demonstrated that obese patients experience higher rates of hospital mortality, low cardiac output syndrome, pulmonary complications, and longer ICU stays than non-obese patients (3, 9, 10). Other studies have demonstrated more frequent sternal wound infections, prolonged ICU stays, and prolonged intubation associated with obesity, but no increase in in-hospital mortality (2, 4, 11). These studies demonstrate mixed findings with some showing a protective effect of Obesity Class I, and others not (1–4, 9, 11, 12).

Despite growing literature, data regarding the relationship between obesity class and postoperative outcomes in patients undergoing surgical management of ATAAD remains conflicting. To address this concern, this study examines the influence of obesity on key clinical outcomes after ATAAD repair in a high-volume aortic center, including in-hospital mortality, stroke, renal failure, prolonged mechanical ventilation, and overall length of hospital stay. We aim to add to the evidence base on ATAAD surgery in obese patients by presenting short-term outcomes of ATAAD surgery from our high-volume cardiovascular institute. By elucidating the potential effects of obesity on ATAAD surgical outcomes, we can better risk stratify patients and inform perioperative planning for this challenging patient population.

## Methods

### Ethical statement

This study was ethically approved by the Northwell Institutional Review Board (IRB No. 220995-NH). The need for patient consent for this retrospective study was waived as part of the IRB approval process. No patient identifiable information is contained.

### Patient population and data collection

The prospectively maintained institutional aortic surgery database was used to retrospectively obtain patient demographics, clinical characteristics, and in-hospital outcomes for all adult patients undergoing ATAAD repair at Lenox Hill Hospital and North Shore University Hospital between July 2014 and December 2022 (*n* = 398). Patients with previous cardiac surgery were excluded, given the added complexity of redo sternotomy. All eligible patients during this study period had a documented height and weight and/or BMI. These patients were stratified into three groups, based on body mass index (BMI), non-obese (BMI < 30 kg/m^2^), Obesity Class I (30 kg/m^2^ ≤ BMI < 35 kg/m^2^), and Obesity Class II + (BMI ≥ 35 kg/m^2^). Obesity Class II (BMI 35–39.9 kg/m^2^) and Obesity Class III (BMI ≥ 40 kg/m^2^) were merged into “Obesity Class II+” group, matching prior studies examining BMI and outcomes in aortic dissection surgery because of the low number of patients and events (2, 10, 11). Preoperative diagnosis was confirmed by CT angiography, with transesophageal echocardiography performed intraoperatively. Patient information is securely maintained in an institutional STS-style registry.

Patient operations were classified into type of aortic repair: isolated ascending, composite valve-graft conduit root replacement (with bioprosthetic or mechanical valve), valve-sparing root replacement, total arch replacement, and frozen elephant trunk. Operation selection and variation of above operations was performed at the discretion of the operating surgeon given dissection pathology, individual patient factors, and aortic anatomy. Intraoperative details such as cardiopulmonary bypass time, aortic cross-clamp time, circulatory arrest time, presence and type of cerebral perfusion used, and arterial cannulation strategy were all recorded, as well as BMI classification per patient.

### Outcomes and statistical analysis

Primary outcome measure was 30-day operative mortality. Secondary outcome measures of intraoperative blood product use, stroke, duration of ventilator support, postoperative kidney injury, duration of ICU stay, bleeding requiring reoperation, deep sternal wound infection, and hospital length of stay were all evaluated.

Descriptive statistics including the frequency distribution for categorical variables and mean, median, SD, interquartile range, minimum, and maximum for continuous variables were calculated to assess baseline patient and operative characteristics. The association between each categorical variable and BMI group was examined using the Chi-square test or Fisher's exact test, as appropriate, to detect differences in the distribution of categorical variables between study cohorts. The association between each continuous variable and BMI group was examined using analysis of variance (ANOVA) or Kruskal–Wallis tests, as appropriate. For categorical and continuous comparisons across the three BMI groups, the *p*-values reported in tables and text were derived from the omnibus test performed. The Dwass-Steel-Critchlow-Fligner multiple comparison test was used to compare the continuous variables between each two groups.

Multivariable logistic regression was utilized to assess the relationship between BMI and 30-day mortality, as well as BMI and stroke. All potential covariates found to be significantly associated with stroke and 30-day mortality in the univariate analysis for each endpoint at a 10% level of significance were entered in a fully saturated multivariable logistic regression model. Backward elimination was used to select the final model, with alpha level set to 5%.

Multivariable linear regression was utilized to assess the relationship between BMI and ventilation time. The outcome ventilation time was log transformed to meet the statistical assumption. Factors found to be significantly associated with ventilation time in the univariate analysis for each endpoint at a 10% level of significance [including prior myocardial infarction, prior stroke, malperfusion syndrome on presentation, age, cardiopulmonary bypass time, and units of fresh frozen plasma (FFP) transfused intraoperatively] were entered in a fully saturated multivariable linear regression model. Variance inflation factor (VIF) was calculated to check for collinearity between variables and all VIF < 2, indicating no collinearity. Backward elimination was used to select the final model, with alpha level set to 5%. The level of significance was set at 5% unless stated otherwise. All aims were analyzed using SAS software Version 9.4 (Cary, NC).

Clinically relevant covariates, including demographic characteristics, comorbidities, markers of presentation severity, and operative variables, were considered for inclusion in multivariable models. Variable selection was guided by clinical relevance and univariate association, with attention to minimizing overfitting relative to the number of outcome events. Model assumptions were assessed.

## Results

### Patient demographics and operative characteristics

A total of 398 patients in our cohort underwent ATAAD repair, 248 (62.3%) of whom were classified as non-obese, 81 (20.4%) as Obesity Class I, and 69 (17.3%) as Obesity Class II + . The preoperative characteristics are listed in Table 1. At baseline, all groups were comparable in terms of sex, hypertension, tobacco use, presence of dyslipidemia, chronic lung disease, chronic renal insufficiency, ejection fraction, Marfan syndrome, bicuspid aortic valve, and most other characteristics. The Obesity Class II + cohort was significantly younger (53.6 ± 14.2) than the non-obese and Obesity Class I cohorts (62.3 ± 13.5 and 61.1 ± 13.0, respectively; *p* < 0.001). The Obesity Class II + cohort was also significantly more likely to have diabetes (23.2%) than the non-obese and Obesity Class I cohorts (10.9% and 9.9%, respectively; *p* = 0.017). Interestingly, the non-obese cohort was significantly more likely to present with signs of shock on presentation (15.3%) than the Obesity Class I and II + cohorts (6.2% and 5.8%, respectively; *p* = 0.020).

**Table 1:** Preoperative characteristics.

Preoperative characteristics	Total	Non-obese	Obesity class I	Obesity class II+	*p*-value
	** (*N*** **=** **398)**	** (*N*** **=** **248)**	** (*N*** **=** **81)**	** (*N*** **=** **69)**	
Female sex	128 (32.2)	76 (30.6)	31 (38.3)	21 (30.4)	0.420
Age, mean ± SD	60.6 ± 13.9	62.3 ± 13.5	61.1 ± 13.0	53.6 ± 14.2	<0.001*
BMI, median (IQR)	28.2 (25.3, 32.5)	26.0 (23.0, 27.7)	31.9 (30.8, 33.4)	38.9 (36.4, 41.9)	<0.001*
Hypertension	308 (77.4)	184 (74.2)	65 (80.2)	59 (85.5)	0.110
Tobacco use	163 (41.0)	103 (41.5)	30 (37.0)	30 (43.5)	0.690
Dyslipidemia	131 (32.9)	83 (33.5)	27 (33.3)	21 (30.4)	0.890
Chronic lung disease	83 (20.9)	56 (22.6)	12 (14.8)	15 (21.7)	0.320
Renal failure requiring dialysis	10 (2.5)	9 (3.6)	0 (0.0)	1 (1.4)	0.190
Diabetes	51 (12.8)	27 (10.9)	8 (9.9)	16 (23.2)	0.017*
Peripheral vascular disease	183 (46.0)	122 (49.2)	38 (46.9)	23 (33.3)	0.064
Cerebral vascular disease	54 (13.6)	28 (11.3)	13 (16.0)	13 (18.8)	0.210
Ejection Fraction	60.0 (55.0, 64.0)	60.0 (55.0, 65.0)	60.0 (55.0, 65.0)	59.0 (55.0, 63.0)	0.870
Prior myocardial infarction	28 (7.0)	18 (7.3)	6 (7.4)	4 (5.8)	0.920
Previous cardiac surgery	0 (0.0)	0 (0.0)	0 (0.0)	0 (0.0)	
Illicit drug use	22 (5.5)	17 (6.9)	3 (3.7)	2 (2.9)	0.440
Bicuspid aortic valve	8 (2.0)	4 (1.6)	4 (4.9)	0 (0.0)	0.081
Marfan Syndrome	9 (2.3)	7 (2.8)	1 (1.2)	1 (1.4)	0.890
Hypertensive on presentation	119 (29.9)	73 (29.4)	22 (27.2)	24 (34.8)	0.580
Normotensive on presentation	185 (46.5)	112 (45.2)	41 (50.6)	32 (46.4)	0.690
Hypotensive on presentation	47 (11.8)	25 (10.1)	13 (16.0)	9 (13.0)	0.330
Shock on presentation (regardless of blood pressure)	47 (11.8)	38 (15.3)	5 (6.2)	4 (5.8)	0.020*
Presence of malperfusion syndrome on presentation	121 (30.4)	66 (26.6)	31 (38.3)	24 (34.8)	0.100
Ischemic peripheral neuropathy	2 (0.5)	2 (0.8)	0 (0.0)	0 (0.0)	>0.999
Stroke	10 (2.5)	6 (2.4)	1 (1.2)	3 (4.3)	0.520
Coma and/or altered consciousness	20 (5.0)	9 (3.6)	8 (9.9)	3 (4.3)	0.087
Mesenteric ischemia	33 (8.3)	19 (7.7)	11 (13.6)	3 (4.3)	0.100
Lower extremity ischemia	78 (19.6)	43 (17.3)	21 (25.9)	14 (20.3)	0.240

Values expressed are *n* (%), % of patients, or median (IQR), unless otherwise specified. *P*-values listed are from omnibus testing.

The asterisks (*) indicate that *p* < 0.05 also known as the comparison reaches statistical significance.

The procedural characteristics of these groups are summarized in Table 2 and Table 3. All groups had comparable frequencies of aortic procedures performed. All groups demonstrated similar cardiopulmonary bypass (CPB), aortic cross-clamp, and circulatory arrest times. Cerebral perfusion strategies and arterial cannulation sites were also not significantly different across groups.

**Table 2:** Aortic procedures performed.

Aortic procedure performed	Total	Non-obese	Obesity class I	Obesity class II+	*p* value
	**(*N*** **=** **398)**	**(*n*** **=** **248)**	**(*n*** **=** **81)**	**(*n*** **=** **69)**	
Isolated replacement of ascending aorta	232 (58.3)	140 (56.5)	48 (59.3)	44 (63.8)	0.540
Aortic root replacement with composite valve-graft conduit	147 (36.9)	95 (38.3)	27 (33.3)	25 (36.2)	0.720
Bioprosthetic	130 (32.7)	85 (34.3)	25 (30.9)	20 (29.0)	0.660
Mechanical	17 (4.3)	11 (4.4)	2 (2.5)	4 (5.8)	0.680
Valve-sparing aortic root replacement	9 (2.3)	7 (2.8)	2 (2.5)	0 (0.0)	0.550
Total arch replacement	29 (7.3)	17 (6.9)	9 (11.1)	3 (4.3)	0.260
Frozen elephant trunk	44 (11.1)	27 (10.9)	7 (8.6)	10 (14.5)	0.520

Values expressed are *n* (%), % of patients, or median (IQR), unless otherwise specified. *P*-values listed are from omnibus testing.

**Table 3:** Operative data.

Operative data	Total	Non-obese	Obesity class I	Obesity class II+	*p* value
	**(*N*** **=** **398)**	**(*n*** **=** **248)**	**(*n*** **=** **81)**	**(*n*** **=** **69)**	
Cardiopulmonary bypass time, minutes, median (IQR)	189.0 (153.0, 244.0)	196.0 (153.8, 249.3)	185.0 (159.0, 225.0)	183.0 (152.0, 241.0)	0.690
Aortic cross-clamp time, minutes, median (IQR)	101.0 (75.0, 143.0)	106.5 (77.0, 149.3)	95.0 (70.0, 131.0)	94.0 (74.0, 145.0)	0.170
Circulatory arrest time, minutes, median (IQR)	27.0 (18.3, 35.0)	28.0 (18.0, 34.3)	28.0 (19.0, 36.0)	24.0 (18.0, 34.0)	0.640
Cerebral perfusion used	246 (61.8)	158 (63.7)	45 (55.6)	43 (62.3)	0.420
Antegrade	208 (52.3)	136 (54.8)	36 (44.4)	36 (52.2)	0.270
Retrograde	9 (2.3)	6 (2.4)	3 (3.7)	0 (0.0)	0.350
Both	29 (7.3)	16 (6.5)	6 (7.4)	7 (10.1)	0.460
Arterial cannulation site					
Femoral	106 (26.6)	70 (28.2)	21 (25.9)	15 (21.7)	0.550
Axillary	128 (32.2)	85 (34.3)	23 (28.4)	20 (29.0)	0.510
Aortic	135 (33.9)	80 (32.3)	28 (34.6)	27 (39.1)	0.560
Innominate	25 (6.3)	9 (3.6)	9 (11.1)	7 (10.1)	0.063

Values expressed are *n* (%), % of patients, or median (IQR), unless otherwise specified. *P*-values listed are from omnibus testing.

Notably, patients in the non-obese cohort were more likely to present with clinical shock, whereas patients in the Obesity Class II + cohort were significantly younger. These baseline differences may reflect variation in presentation severity across BMI groups.

### Unadjusted outcomes

The unadjusted outcomes are presented in Table 4 and Table 5. Intraoperative packed red blood cells (pRBCs) were significantly more likely to be used and more units were used in the non-obese cohort than the Obesity Class I and Obesity Class II + patients (*p* < 0.001). Patients in the non-obese cohort utilized significantly more units of fresh frozen plasma and platelets than did those in the Obesity Class I or II + groups (*p* = 0.036 and 0.024, respectively).

**Table 4:** Intraoperative transfusion requirements.

Transfusion requirements	Total	Non-obese	Obesity class I	Obesity class II+	*p* value
	**(*N*** **=** **398)**	**(*n*** **=** **248)**	**(*n*** **=** **81)**	**(*n*** **=** **69)**	
Intraoperative use of packed red blood cells (PRBCs)	270 (67.8)	186 (75.0)	48 (59.3)	36 (52.2)	<0.001*
Intraoperative use of PRBCs, median # of units (IQR)	2.0 (0.0, 4.0)	2.0 (0.8, 4.0)	1.0 (0.0, 3.0)	1.0 (0.0, 2.0)	<0.001*
Intraoperative use of fresh frozen plasma (FFP)	280 (70.4)	183 (73.8)	51 (63.0)	46 (66.7)	0.140
Intraoperative use of FFP, median # of units (IQR)	2.0 (0.0, 3.0)	2.0 (0.0, 4.0)	2.0 (0.0, 2.0)	2.0 (0.0, 2.0)	0.036*
Intraoperative use of platelets	346 (86.9)	221 (89.1)	68 (84.0)	57 (82.6)	0.250
Intraoperative use of platelets, median # of units (IQR)	2.0 (1.0, 2.0)	2.0 (1.0, 3.0)	2.0 (1.0, 2.0)	2.0 (1.0, 2.0)	0.024*
Intraoperative use of cryoprecipitate	304 (76.4)	192 (77.4)	57 (70.4)	55 (79.7)	0.330
Intraoperative use of cryoprecipitate, median # of units (IQR)	5.0 (5.0, 10.0)	5.0 (5.0, 10.0)	5.0 (0.0, 10.0)	5.0 (5.0, 10.0)	0.330

Values expressed are *n* (%), % of patients, or median (IQR), unless otherwise specified. *P*-values listed are from omnibus testing.

The asterisks (*) indicate that *p* < 0.05 also known as the comparison reaches statistical significance.

**Table 5:** Postoperative outcomes.

Postoperative outcomes	Total	Non-obese	Obesity class I	Obesity class II+	*p* value
	**(*N*** **=** **398)**	**(*n*** **=** **248)**	**(*n*** **=** **81)**	**(*n*** **=** **69)**	
30-day mortality	51 (12.8)	38 (15.3)	4 (4.9)	9 (13.0)	0.052
Postoperative stroke	54 (13.6)	31 (12.5)	14 (17.3)	9 (13.0)	0.550
Bleeding requiring reoperation	24 (6.0)	14 (5.6)	5 (6.2)	5 (7.2)	0.830
Deep sternal wound infection	2 (0.5)	1 (0.4)	0 (0.0)	1 (1.4)	0.360
New onset dialysis	90 (22.6)	55 (22.2)	15 (18.5)	20 (29.0)	0.300
Sepsis	29 (7.3)	21 (8.5)	2 (2.5)	6 (8.7)	0.170
Ventilation hours, median (IQR)	25.3	25.3	30	20.8	0.780
	(12.0, 111.0)	(11.9, 100.0)	(14.0, 121.8)	(11.3, 128.0)	
ICU duration, hours, median (IQR)	120.5	117.5	130	132	0.180
	(67.6, 257.5)	(65.9, 211.6)	(72.0, 266.3)	(62.0, 326.0)	
Length of stay, days, median (IQR)	11	11	11	10	0.560
	(7.0, 19.0)	(7.0, 18.0)	(7.0, 21.0)	(6.0, 20.0)	

Values expressed are *n* (%), % of patients, or median (IQR), unless otherwise specified. *P*-values listed are from omnibus testing.

Overall, 30-day operative mortality for the sample was 51/398 (12.8%). The non-obese group had a rate of 38/248 (15.3%), the Obesity Class I group had a rate of 4/81 (4.9%) and the Obesity Class II + group had a rate of 9/69 (13.0%). While these differences trend toward Obesity Class I status as a protective factor, this difference did not reach significance (*p* = 0.052). Other postoperative outcomes including stroke, bleeding requiring reoperation, new onset renal failure requiring dialysis, and sepsis occurred at similar rates across all groups. Ventilation, ICU and overall length of stay times were also not significantly different across all groups.

### Multivariable analysis

According to the multivariable analysis, the Obesity Class I group was found to be associated with decreased odds of 30-day operative mortality [OR = 0.18, 95% CI: (0.06–0.62), *p* = 0.006] with reference to the non-obese group while adjusting for other factors (including hemodynamic status) in the model. The Obesity Class II + group did not demonstrate a significantly different rate of 30-day operative mortality when compared with the non-obese group [OR = 1.03, 95% CI: (0.42–2.53), *p* > 0.9]. Regardless of obesity class, patients with diabetes [OR = 2.9, 95% CI: (1.32–6.41), *p* = 0.008], those who presented with lower extremity ischemia [OR = 4.27, 95% CI: (2.11–8.62), *p* < 0.001], those who were older [OR = 1.04, 95% CI: (1.01–1.07), *p* = 0.005], had longer CPB time [OR = 1.005, 95% CI: (1.00–1.01), *p* = 0.027] and those who received more units of intraoperative pRBCs [OR = 1.08, 95% CI: (1.01–1.16), *p* = 0.028] were more likely to experience 30-day mortality, independent of other factors in the analysis.

Further, this patient cohort demonstrated that patients in Obesity Class I or II + did not demonstrate a significantly higher rate of stroke in the postoperative period than did non-obese patients [Obesity Class I OR = 1.30, 95% CI: [0.67–2.86], *p* = 0.400; Obesity Class II + OR = 1.28, 95% CI: [0.56–2.89], *p* = 0.600]. Regardless of obesity class, those who presented in a coma or with significantly altered consciousness [OR = 2.81, 95% CI: (1.003–7.85), *p* = 0.049], and those who received a higher number of units of pRBCs intraoperatively [OR = 1.10, 95% CI: (1.03–1.17), *p* = 0.002] were significantly more likely to develop a postoperative stroke than others, independent of other factors in the analysis.

Finally, there was no significant association between obesity class and post-operative ventilation time [Obesity Class I beta = 0.023, 95% CI: [−0.312–0.358], *p* = 0.900; Obesity Class II + beta = 0.171, 95% CI: [−0.198–0.541], *p* = 0.400]. Patients with a history of stroke [beta = 0.524, 95% CI: (0.139–0.909), *p* = 0.008], prior myocardial infarction [beta = 0.605, 95% CI: (0.072–1.14), *p* = 0.026], increased age [beta = 0.018, 95% CI: (0.008–0.028), *p* < 0.001], longer CPB time [beta = 0.003, 95% CI: (0.001–0.005), *p* < 0.001], malperfusion syndrome at presentation [beta = 0.449, 95% CI: (0.156–0.742), *p* = 0.003], and greater intraoperative fresh frozen plasma transfusion [beta = 0.103, 95% CI: (0.037–0.168), *p* = 0.002] were significantly associated with longer duration of ventilation support, independent of other factors in the analysis.

## Discussion

Optimal management of ATAAD in obese patients requires clarification as obesity continues to increase worldwide and is strongly associated with cardiovascular disease (13). Our retrospective study of ATAAD patients undergoing operative repair demonstrated reduced risk of 30-day mortality associated with class I obesity compared with non-obesity. Although counterintuitive, this finding aligns with previous cardiac surgery literature supporting the “obesity paradox.”

The obesity paradox refers to the observation that overweight and mildly obese patients often demonstrate better short-term survival rates and fewer complications following cardiac surgery compared to their healthy-weight or underweight counterparts, despite obesity being a well-established risk factor for cardiovascular disease. This paradox yields a U-shaped relationship between BMI and mortality, possibly owing to nutritional reserve, decreased frailty, and lack of cardiac cachexia (2, 9, 14–16). It is also theoretically possible that obese patients may receive more aggressive medical management and closer monitoring due to their perceived higher risk, potentially leading to better outcomes.

Previous small studies which evaluated outcomes of obese patients after surgery for ATAAD demonstrated that obese patients experienced higher rates of hospital mortality, low cardiac output syndrome, pulmonary complications, longer ventilator dependence times, and longer ICU stays than non-obese patients (3, 9, 10). Other studies have demonstrated more frequent sternal wound infections, prolonged ICU stays, and prolonged intubation associated with obesity, but not increased in-hospital mortality (2, 4, 11). This study does not demonstrate significantly increased rates of sternal wound infections, prolonged ICU stays, or prolonged intubation. This study demonstrated reduced adjusted 30-day mortality in Obesity Class I patients compared to non-obese patients. However, Obesity Class II and non-obese patients had similar rates of 30-day mortality. This study demonstrates a U-shaped relationship in operative mortality after ATAAD repair as BMI increases, previously described as the obesity paradox (15).

It is important to emphasize that these findings should not be interpreted as promoting or endorsing obesity in patients with cardiovascular pathology as there is well-established evidence demonstrating the negative impacts of obesity on overall health. A more appropriate interpretation is that there is a complex relationship between body composition and surgical outcomes that is not fully understood (17). While in an ideal world, individualized risk assessment and pre-operative nutrition optimization would be done in all patients before ATAAD repair, the hyperacute and emergent nature of the pathology often prevents optimization, so evidence supporting the safety of operative management in patients regardless of BMI is reassuring.

The long-term implications of the obesity paradox also warrant careful consideration. While short-term outcomes may appear favorable for overweight and mildly obese patients, the long-term consequences of excess body weight on cardiovascular health and overall mortality remain a significant concern. Studies have suggested that the apparent protective effect diminishes over time, highlighting the need for extended follow-up periods in future research (2, 11).

While class I obesity was associated with lower adjusted odds of 30-day mortality, this finding should be interpreted cautiously. This study's unadjusted 30-day mortality comparison yielded a non-significant difference, though there was a strong signal towards reduced mortality with Obesity Class I this comparison wasn't significant until multivariable logistic regression was performed. Given the observational design and baseline differences between groups, including variations in presentation acuity, this association does not imply a causal protective effect and should be considered hypothesis-generating.

Future research directions should focus on elucidating the underlying mechanisms responsible for the obesity paradox, including potential protective factors associated with adipose tissue and the role of muscle mass in surgical outcomes. Additionally, prospective studies with longer follow-up periods are needed to better understand the long-term implications of this phenomenon.

### Limitations

It is crucial to consider potential confounding factors and limitations in interpreting these results. As a retrospective cohort study, the causal inferences that can be drawn from the results are limited. Further, this study takes place at two high-volume aortic surgery centers, therefore while these findings may be most directly applicable to obese patients at similar high volume referral centers for aortic surgery, these findings may be less generalizable to lower volume centers with less experience managing ATAAD in this population. Additionally, our study does not include long-term follow up data so it is not possible to compare long-term outcomes of ATAAD repair in this population. The use of BMI as the sole measure of obesity fails to account for body composition, particularly the distribution of fat and muscle mass. Future studies incorporating more comprehensive measures of body composition, such as waist-to-hip ratio or body fat percentage, may provide more nuanced insights into this relationship. Future studies could also investigate markers of nutritional status (albumin, prealbumin, recent weight changes) and how they are predictive of post-operative outcomes in this patient population.

Furthermore, selection bias may play a role in the observed paradox. Severely obese patients are sometimes excluded from cardiac surgery due to perceived high risk, potentially skewing the study population towards those with better overall health status within the obese category.

## Conclusion

The obesity paradox in cardiac surgery remains a complex and intriguing area of study. While our findings support paradoxically improved mortality outcomes in patients with Obesity Class I, there is a need for further study into the role of pre-operative body composition and nutritional status in pre-operative risk stratification. Further investigation into long-term outcomes after ATAAD surgical repair and how the obese patient population's long-term outcomes compare to those of non-obese patients will be critical going forward. This study has found that obesity does not significantly worsen operative 30-day mortality rates in patients undergoing ATAAD repair. Expeditious surgical intervention is still recommended for all primary ATAAD, irrespective of BMI.

## Data Availability

The raw data supporting the conclusions of this article will be made available by the authors, without undue reservation.
